# An Ultrasonic-Based Radiomics Nomogram for Distinguishing Between Benign and Malignant Solid Renal Masses

**DOI:** 10.3389/fonc.2022.847805

**Published:** 2022-03-04

**Authors:** Chunxiang Li, Ge Qiao, Jinghan Li, Lisha Qi, Xueqing Wei, Tan Zhang, Xing Li, Shu Deng, Xi Wei, Wenjuan Ma

**Affiliations:** ^1^ Department of Diagnostic and Therapeutic Ultrasonography, Tianjin Medical University Cancer Institute and Hospital, Tianjin, China; ^2^ National Clinical Research Center for Cancer, Tianjin, China; ^3^ Key Laboratory of Cancer Prevention and Therapy, Tianjin, China; ^4^ Tianjin’s Clinical Research Center for Cancer, Tianjin, China; ^5^ Department of Pathology, Tianjin Medical University Cancer Institute and Hospital, Tianjin, China; ^6^ Department of Diagnostic and Therapeutic Ultrasonography, Tianjin Ninghe Hospital, Tianjin, China; ^7^ Second Hospital of Tianjin Medical University, Tianjin, China; ^8^ Department of Breast Imaging, Tianjin Medical University Cancer Institute and Hospital, Tianjin, China

**Keywords:** renal mass, angiomyolipoma, oncocytoma, ultrasound, radiomics

## Abstract

**Objectives:**

This study was conducted in order to develop and validate an ultrasonic-based radiomics nomogram for diagnosing solid renal masses.

**Methods:**

Six hundred renal solid masses with benign renal lesions (*n* = 204) and malignant renal tumors (*n* = 396) were divided into a training set (*n* = 480) and a validation set (*n* = 120). Radiomics features were extracted from ultrasound (US) images preoperatively and then a radiomics score (RadScore) was calculated. By integrating the RadScore and independent clinical factors, a radiomics nomogram was constructed. The diagnostic performance of junior physician, senior physician, RadScore, and radiomics nomogram in identifying benign from malignant solid renal masses was evaluated based on the area under the receiver operating characteristic curve (ROC) in both the training and validation sets. The clinical usefulness of the nomogram was assessed using decision curve analysis (DCA).

**Results:**

The radiomics signature model showed satisfactory discrimination in the training set [area under the ROC (AUC), 0.887; 95% confidence interval (CI), 0.860–0.915] and the validation set (AUC, 0.874; 95% CI, 0.816–0.932). The radiomics nomogram also demonstrated good calibration and discrimination in the training set (AUC, 0.911; 95% CI, 0.886–0.936) and the validation set (AUC, 0.861; 95% CI, 0.802–0.921). In addition, the radiomics nomogram model showed higher accuracy in discriminating benign and malignant renal masses compared with the evaluations by junior physician (DeLong *p* = 0.004), and the model also showed significantly higher specificity than the senior and junior physicians (0.93 vs. 0.57 vs. 0.46).

**Conclusions:**

The ultrasonic-based radiomics nomogram shows favorable predictive efficacy in differentiating solid renal masses.

## Introduction

Although the majority of renal masses are malignant, about 16% to 19% of renal tumors are reported to be benign (1–3). Clinically, renal cancers need to be surgically resected, whereas for benign renal neoplasms, especially for small renal masses, conservative management is performed. Unlike other cancers, there are currently no serum biomarkers available to confirm the identity of renal masses. Accurate preoperative identification of benign from malignant solid renal masses is challenging for a radiologist (4).

Percutaneous renal biopsy is an important pretreatment diagnostic procedure in the evaluation of indeterminate renal masses. However, the diagnostic accuracy of percutaneous biopsy ranges from 70% to 90%, and its role in clinical management remains unclear because of the negative predictive value and the possible complications, including bleeding, perirenal hematoma, hematuria, arteriovenous fistula formation, and pneumothorax (5–7). Thus, it is of vital importance to search for an accurate as well as safe and non-invasive diagnostic tool to distinguish benign from malignant solid renal masses in the preoperative clinical decision-making process.

Conventional B-mode ultrasound (US) is an easy, safe, and non-invasive procedure. It is currently the first-line imaging modality for detecting renal lesions and discriminating benign from malignant renal tumors. However, the visual interpretation of US images is generally based on the sonographers’ experience. Radiomics features can be computed from grayscale images to reflect the texture and morphology of tumors (8–11). Based on US images, radiomics has been applied to evaluate various tumors, including hepatocellular carcinomas, breast cancer, and thyroid carcinomas (12–15). However, to the best of our knowledge, no published study has applied ultrasonic-based radiomics to renal tumors. The purpose of this study was to develop and validate an ultrasonic-based radiomics nomogram for the differentiation between benign and malignant solid renal masses.

## Material and Methods

### Study Cohort and Imaging Dataset

**Figure 1:**
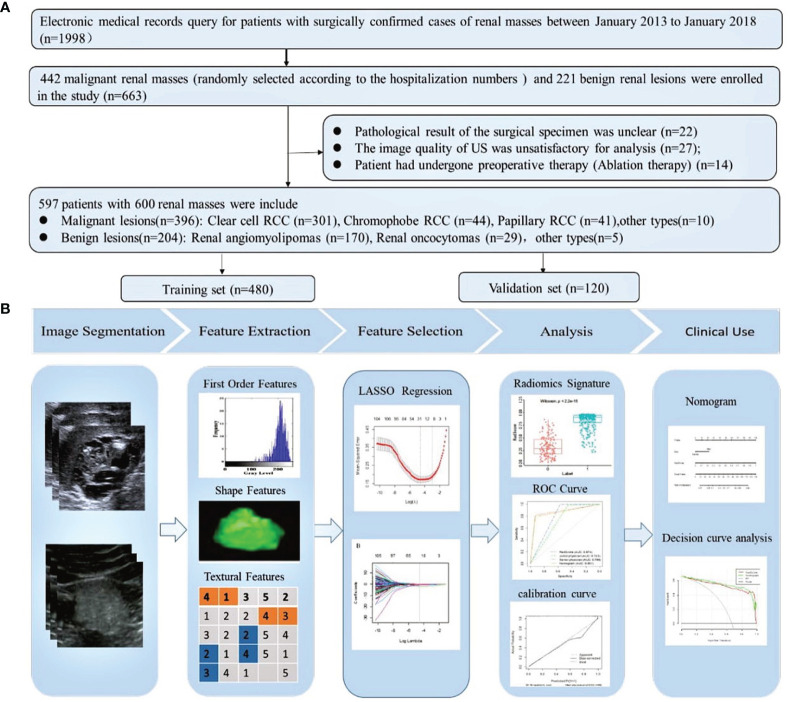
**(A)** Flowchart of the inclusion, exclusion, and grouping criteria for patients with renal masses. One patient had three clear cell RCCs. One patient had two clear cell RCCs. RCC, renal cell carcinoma. **(B)** Flowchart of the radiomics analysis of renal masses.

We queried our institution’s electronic medical records to derive all surgically confirmed cases of renal masses between January 2013 and January 2018. A total of 1,998 confirmed renal masses were screened in this query: 1,777 malignant renal lesions and 221 benign renal lesions. In order to balance the proportion of malignant and benign renal masses, we randomly selected 442 malignant renal masses according to the hospitalization numbers. The inclusion criteria were as follows: 1) patients who had undergone US examination before treatment and the image quality of US was satisfactory for analysis and 2) patients with complete clinicopathological data. The exclusion criteria were as follows: 1) the pathological result of the surgical specimen was unclear, 2) the patient had undergone preoperative therapy (ablation therapy), and 3) the renal mass was not completely visible in the image. Finally, 597 patients with 600 renal masses were included in this study (**Figure 1A**). A summary of the patient characteristics is presented in **Supplementary Material 1**. The enrolled renal masses in this study were divided randomly into a training set (*n* = 480) and a validation set (*n* = 120). The distribution of tumors is detailed in **Table 1**. This study was approved by the Ethics Committee of Tianjin Medical University Cancer Institute and Hospital and informed consent was waived.

**Table 1:** Clinical characteristics of patients in the training and validation sets.

Characteristics	Training set (*N* = 480)						Validation set (*N* = 120)			
	Benign	Malignant	Univariate analysis	Multivariate analysis			Benign		Malignant	Univariate analysis
	(*n* = 161)	(*n* = 319)	*p*	OR (95% CI)	*p*		(*n* = 43)		(*n* = 77)	*p*
Sex			<0.001		<0.001					<0.001
Male	31 (19.3)	206 (64.6)		0.848 (0.804–0.894)			10 (23.3)		59 (76.6)	
Female	130 (80.8)	113 (35.4)					33 (76.7)		18 (23.4)	
Age			<0.001		0.0544					0.603
<53	102 (63.4)	136 (42.6)					20 (46.5)		32 (41.6)	
≥53	59 (36.7)	183 (57.4)					23 (53.5)		45 (58.4)	
Symptoms			0.577							0.667
No	136 (84.5)	263 (82.5)					36 (83.7)		62 (80.5)	
Yes	25 (15.5)	56 (17.6)					7 (16.3)		15 (19.5)	
Location			0.289							0.746
Right	75 (46.6)	165 (51.7)					21 (48.8)		40 (52.0)	
Left	86 (53.4)	154 (48.3)					22 (51.2)		37 (48.1)	
Size (cm)			0.871							0.363
≤4	90 (55.9)	171 (53.6)					23 (53.5)		36 (46.8)	
>4, ≤7	53 (33.9)	112 (35.1)					17 (39.5)		32 (41.6)	
>7	18 (11.2)	36 (11.3)					3 (7.0)		9 (11.7)	
RadScore	0.330 ± 0.201	0.834 ± 0.174	<0.001	3.158 (2.89–3.45)	<0.001			0.347 ± 0.198	0.824 ± 0.204	<0.001

RadScore, radiomics score; OR, odds ratio; CI, confidence interval.

### US Image Acquisition

US examination was performed using the Philips iU22 system (Philips Ultrasound, Bothell, WA, USA) and Aplio 500 (Toshiba Medical Systems, Tokyo, Japan). The C5-1 probe was used for conventional ultrasound with a central frequency of 3–5 MHz. The gray image of the renal masses was available.

### Workflow

The workflow of the radiomics analysis included tumor segmentation, feature extraction, feature selection, and radiomics signature construction and evaluation (**Figure 1B**).

### Image Segmentation, Preprocessing, and Radiomics Feature Extraction

All US images were retrieved from the picture archiving and communication systems (PACS) for image segmentation and analysis in our institution. Lesions were segmented using ImageJ (https://imagej.nih.gov/ij/). A sonographer with more than 8 years of experience in US imaging of kidney neoplasms semiautomatically corrected the boundary of the lesion in each image of each individual patient. When the boundary is not determined, another experienced sonographer (with 20 years of experience in abdominal diagnosis) was consulted for a final opinion.

From each of the segmented objects, we applied existing automated computer programs to extract a set of 855 radiomic features for each patient with renal mass. These features were divided into four groups: 1) morphological features, such as area, largest diameter, length to width ratio, and roundness; 2) grayscale statistic (GSS) features calculated from the histogram of tumor voxel intensities, such as variance, skewness, and kurtosis; 3) texture feature, including gray-level co-occurrence matrix (GLCM), gray-level run-length matrix (GLRLM), gray-level size zone matrix (GLSZM), and neighborhood gray-tone difference matrix (NGTDM); and 4) wavelet feature. Details of the procedures for extraction of radiomic features are described in **Supplementary Material 2**. *Z*-score normalization was performed as preprocessing steps for data to guarantee the repeatability of the results.

### Development of the Radiomics Signature Model

Dimension reduction of the features was conducted to minimize overfitting and reduce the bias from radiomics features in the modeling. Firstly, the Mann–Whitney *U* was used to select features that were highly related to the biomarkers. A significance level of 0.05 (*p* < 0.05) was set as the threshold. Secondly, an interfeature coefficient (*R*) between all possible pairs of features was subsequently used to eliminate high-dimensional feature redundancy. *R >*0.8 was the cutoff for strong relationships, in which one of two features with a lower *p*-value was excluded. Next, the least absolute shrinkage selection operator (LASSO) method was used to select the most important features with non-zero coefficients, and a RadScore was calculated for each patient. The process of radiomics feature extraction and analysis was performed in MATLAB 2018a (The MathWorks Inc., Natick, MA, USA) and R software (version 6.1, R Foundation for Statistical Computing, Vienna, Austria), respectively.

### Development of a Radiomics Nomogram Model and the Performance of Different Models

The significant variables of the clinical factors and RadScore were integrated to build the radiomics nomogram model. We performed a calibration curve to graphically investigate the performance characteristics of the nomogram. The performance of each model for differentiating benign from malignant solid renal masses was evaluated based on the area under the ROC, accuracy, specificity, and sensitivity in both the training and validation sets. The difference in the AUC between the training and validation datasets was tested by the *p*-value of Delong’s test. The clinical usefulness of the nomogram was assessed using DCA which included two decision curves based on the radiomics signature and radiomics nomogram and could be demonstrated by calculating the net benefits for a range of threshold probabilities.

### Image Analysis

The US findings were independently analyzed by two sonographers (XW and CL, with 20 and 5 years of experience, respectively), including size, location, shape, margin, and echogenicity.

### Statistical Methods

The continuous variables were described as the mean ± standard deviation (*x̅* ± *s*). The categorical data were presented as percentage or ratio. The logistic regression method was used for univariate and multivariate analysis in the training set. Statistical significance was set at *p <*0.05.

## Results

### Differences in the Clinical Features Between Benign and Malignant Patients

Detailed information on the clinical characteristics is shown in **Table 1**. There was a significant difference in sex (*p* < 0.001) and age (*p* < 0.001) between benign and malignant patients in the training set. Multiple logistic regression analysis showed that sex (*p* < 0.001, OR: 0.848; 95% CI: 0.804–0.894) remained as an independent predictor.

### Radiomics Feature Selection and Radiomics Signature Model Building

**Figure 2:**
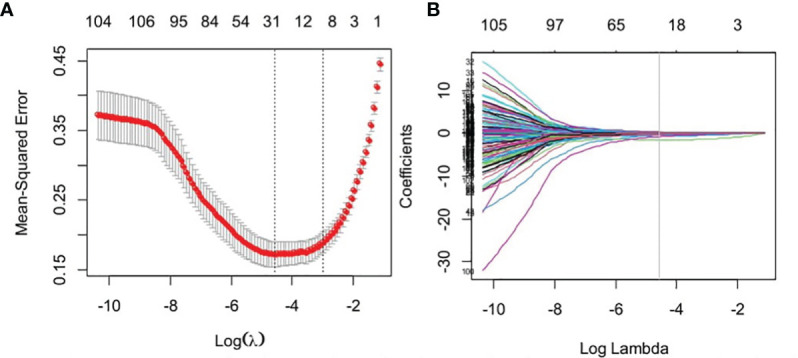
Feature selection using the least absolute shrinkage and selection operator (LASSO) binary logistic regression model. **(A)** Tuning parameter (lambda) selection in the LASSO model used 10-fold cross-validation *via* minimum criteria. **(B)** The gray line in the figure is the partial likelihood estimate corresponding to the optimal value of lambda. The optimal lambda value of 0.061 was chosen.

**Figure 3:**
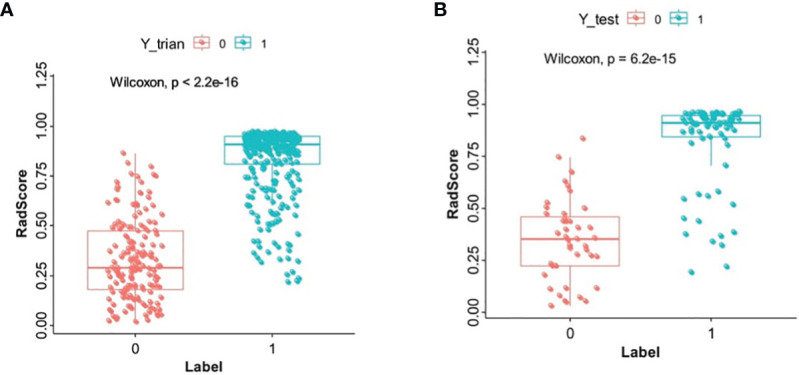
The boxplot of RadScore from the training set **(A)**. The boxplot of RadScore from the validation set **(B)**.

Of the radiomics features, 855 features were reduced to 30 potential predictors on the basis of 480 renal masses in the training set (**Figure 2**), and these features were presented in the RadScore calculation formula (**Supplementary Material 3**). We compared the RadScores from the training and validation sets, respectively (**Figure 3**). The cutoff value was 0.633. There was a significant difference in the distributions of the RadScore of the benign and malignant groups in these two sets.

### Development of a Radiomics Nomogram Model

**Figure 4:**
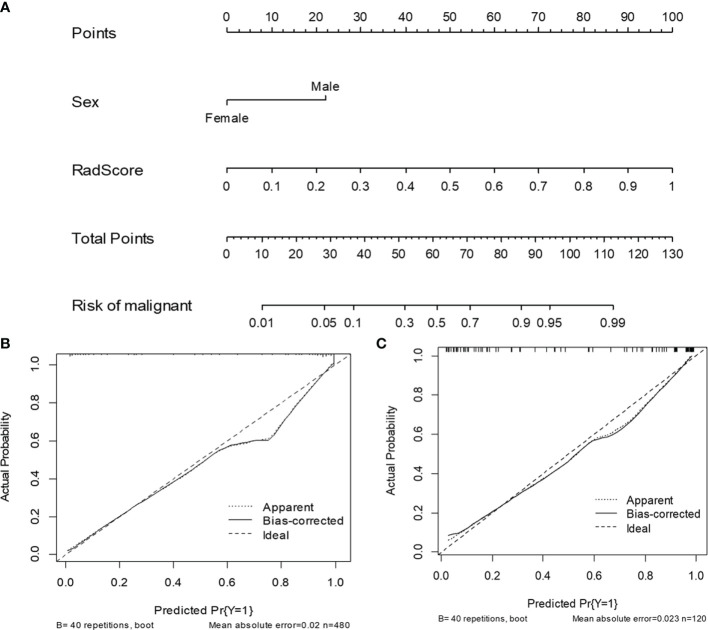
The radiomics nomogram and calibration curves for the radiomics nomogram. The radiomics nomogram, combining sex and RadScore, developed in the training set **(A)**. Calibration curves for the radiomics nomogram in the training **(B)** and validation **(C)** sets.

**Figure 5:**
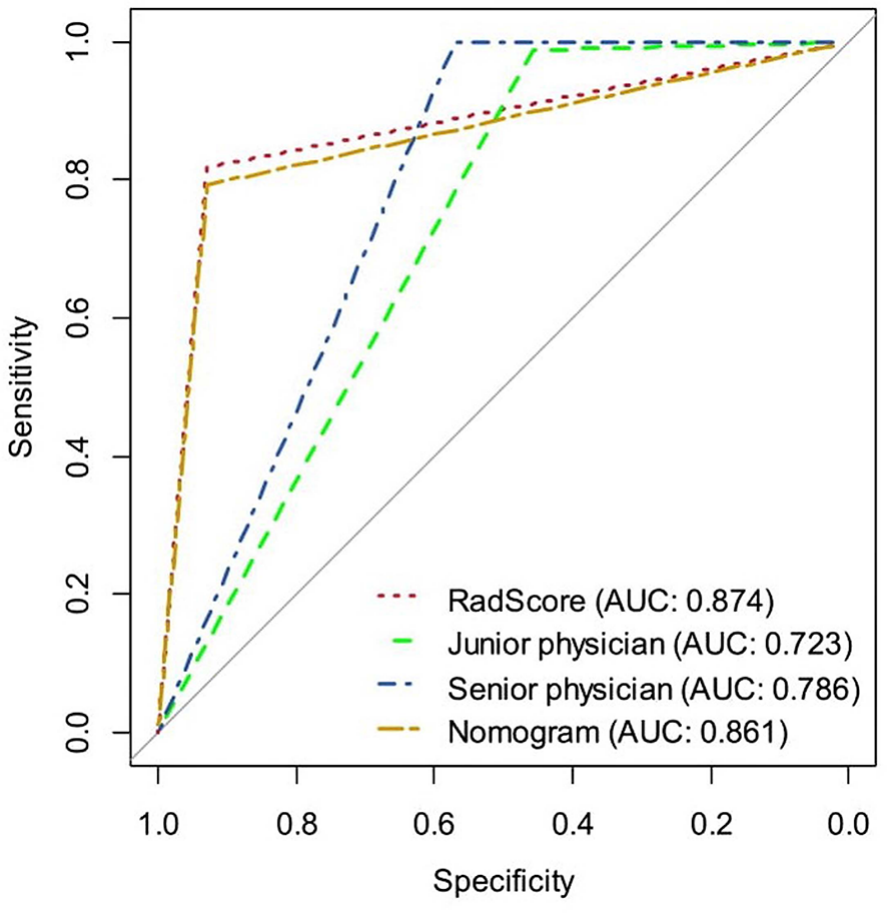
The ROC curves of the radiomics signature, radiomics nomogram, senior physician, and junior physician in the validation set, respectively.

**Table 1** shows the results of the univariate and multivariate logistic regression analyses in the training set. Sex and RadScore were identified as independent factors for predicting the properties of solid renal masses (*p* < 0.001). Thus, the radiomics nomogram was developed using sex and radiomics score (**Figure 4A**). The calibration plots of this radiomics nomogram showed good calibration in the training and validation sets (**Figures 4B, C**). The performance of the radiomics signature model and the radiomics nomogram model for both the training and validation sets is presented in **Table 2**. Although the AUC of the radiomics signature model was slightly higher than that of the radiomics nomogram model, the discriminating ability of the radiomics model was comparable to that of the radiomics nomogram model (**Figure 5**, DeLong test: *p* = 0.221 for the training set, *p* = 0.760 for the validation set).

**Table 2:** Diagnostic performance of radiomics signature, radiomics nomogram, senior physician, and junior physician in the validation set.

	Method	AUC (95% CI)	Accuracy (95% CI)	Sensitivity	Specificity	PPV	NPV
Training set	Radiomics	0.887 (0.860–0.915)	0.873 (0.840, 0.901)	0.843	0.932	0.960	0.750
	Nomogram	0.911 (0.886–0.936)	0.898 (0.867, 0.924)	0.872	0.950	0.972	0.789
Validation set	Radiomics	0.874 (0.816–0.932)	0.858 (0.783, 0.915)	0.818	0.930	0.955	0.741
	Nomogram	0.861 (0.802–0.921)	0.842 (0.764, 0.902)	0.792	0.932	0.953	0.714
	Senior physician	0.786 (0.703–0.869)	0.875 (0.802, 0.928)	1.0	0.571	0.850	1.0
	Junior physician	0.723 (0.638–0.807)	0.833 (0.754, 0.895)	0.988	0.457	0.815	0.941

CI, confidence interval; PPV, positive predictive value; NPV, negative predictive value.

**Figure 6:**
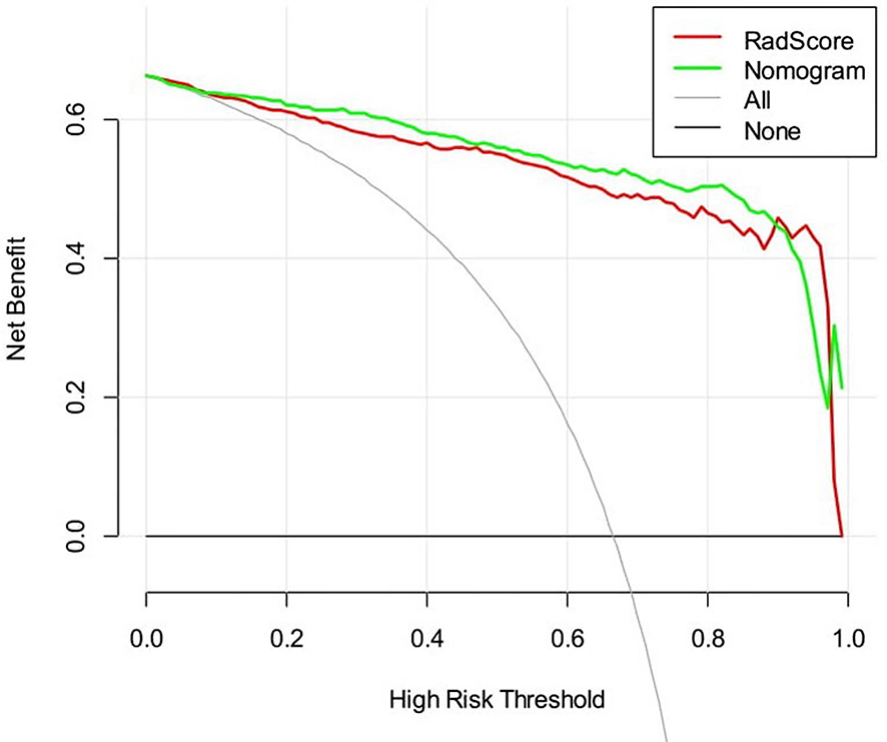
Decision curve analysis for two models. The *y*-axis indicates the net benefit; the *x*-axis indicates threshold probability. The red line and green line represent net benefit of the radiomics signature and the radiomics nomogram, respectively.

The DCAs based on the two models are presented in **Figure 6**. For the differentiation of benign from malignant renal masses, the nomogram model had a higher overall net benefit than the radiomics signature model across the majority of the range of reasonable threshold probabilities.

### The Diagnostic Performance of the Radiomics Signature, Radiomics Nomogram, and Senior and Junior Physicians

**Table 2** and **Figure 5** show the diagnostic performance of the radiomics signature, radiomics nomogram, and senior and junior physicians in the validation set. There was a significant difference between the junior physician and the radiomics nomogram (DeLong *p* = 0.004), but there was no significant difference between the senior physician and the radiomics nomogram (DeLong *p* = 0.089). However, the specificity of the radiomics nomogram was significantly higher than that of the senior and junior physicians (0.93 vs. 0.57 vs. 0.46).

## Discussion

Clinically, for the renal masses that are radiographically suspicious for renal cell carcinoma (RCC) without a pathology diagnosis, nephrectomy or nephron-sparing surgery is a standard management (16, 17). However, approximately 20%–30% of surgically removed renal masses are reported to be benign (1, 4, 18–21), and this may lead to overtreatment of benign renal tumors. Percutaneous needle biopsy is not used in all patients because of the uncertain effect and safety. Thus, there is a great and increasing need for accurate and non-invasive methods to distinguish benign from malignant renal masses before surgery. In this study, by combining US image radiomics and clinical characteristics, we developed a nomogram model that demonstrated good accuracy in differentiating benign from malignant renal masses.

In clinical practice, many imaging methods are used to distinguish between benign and malignant renal masses. US is currently the first-line imaging modality for renal tumor screening. Contrast-enhanced CT is the most commonly used imaging equipment for the evaluation of a renal tumor that requires further observation. With a superior soft-tissue contrast, MRI is particularly helpful to distinguish solid from cystic lesions. Contrast-enhanced US (CEUS) could be used to show the real-time tumor vascularization. Compared with US, these imaging procedures are more expensive, time-consuming, and less safe due to the use of nephrotoxic iodine contrast agents and ionizing radiation. Some reports have indicated the significance of CT- or MRI-based radiomics model in discriminating benign renal tumor from malignancies (22–29). However, there are no US-based radiomic studies used to identify renal tumors to date. Here, based on US images radiomics and combined with clinical characters, we developed a nomogram model that demonstrated good accuracy in differentiating benign from malignant renal masses. 

In the present study, among the 855 extracted radiomics features, 30 features including texture and wavelet features were selected as the significant features to build the radiomics signature. The images in the benign group were more homogeneous in texture than the ones in the RCC group, which was consistent with previous reports and the diagnostic experience of the sonographer (22, 23).

Recently, radiomics showed excellent performance in differentiating benign and malignant renal masses (22–29). Hodgdon et al. developed a model incorporating CT texture features from 100 patients to differentiate angiomyolipomas (AMLs) from RCCs, which resulted in an AUC of 0.89 (22). Said et al. assessed the diagnostic value of MRI-based radiomics features using machine learning (ML) from 104 RCCs and 21 benign lesions, with the best diagnostic performance in the validation sets showing AUC of 0.73 (28). The radiomics model from our study achieved an AUC of 0.87 in the validation set, which was comparable to CT- or MRI-based radiomics. Moreover, the radiomics signature and nomogram model showed excellent diagnostic performance comparable to senior physician, indicating the potential significance of the model in clinical application in the hope that it will advance precision diagnostics, even for the junior physician without a wealth of experience in imaging diagnosis.

**Figure 7:**
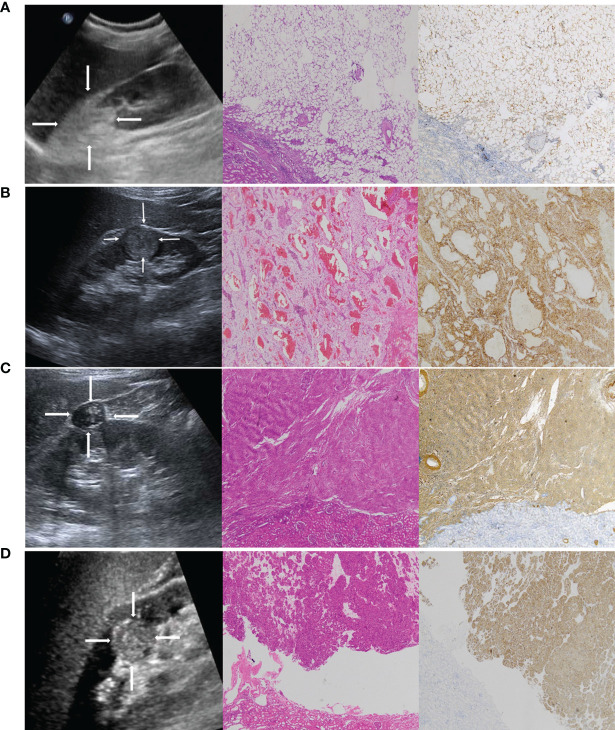
**(A)** A 42-year-old female patient with a strong hyperechoic tumor in the upper pole of the right kidney; the pathology report showed angiomyolipoma (AML). Representative ultrasound image (left). The echogenicity corresponds with a composition of fat. The pathological H&E staining image (middle, ×50) showed that the tumor was mainly composed of fat. S-100 immunohistochemical staining image (right, ×50) was used to label the lipid composition. **(B)** A 65-year-old male patient with a hypoechoic tumor located in the lower middle of the right kidney; the pathology report showed clear cell RCC. Representative ultrasound image (left). The pathological H&E staining image (middle, ×50). CA-IX immunohistochemical staining image (right, ×50) was used to label the clear cell RCC. **(C)** A 51-year-old female patient with a hypoechoic tumor located in the upper middle of the right kidney; the pathology report showed AML without visible fat. It was misdiagnosed as RCC by a sonographer. Representative ultrasound image (left). The pathological H&E staining image (middle, ×50) showed that the tumor was mainly composed of smooth muscle. SMA immunohistochemical staining image (right, ×50) was used to label the smooth muscle composition. **(D)** A 47-year-old female patient with a hypoechoic tumor located in the middle of the right kidney; the pathology report showed oncocytoma. It was misdiagnosed as RCC by a sonographer. Representative ultrasound image (left). The pathological H&E staining image (middle, ×50). CD117 immunohistochemical staining image (right, ×50) was used to label the oncocytoma.

We further analyzed the false diagnosed cases. In general, classic AMLs consist of aberrant blood vessels, smooth muscle, and mature adipose tissue (30). They are markedly hyperechoic due to the presence of macroscopic fat (31, 32) (**Figure 7A**) and easy to distinguish from RCCs, which are more isoechoic or hypoechoic (**Figure 7B**). However, approximately 5% of AML cases lack macroscopic fat (**Figure 7C**), demonstrating sonographic appearances similar to those of RCC (33–35). In addition, most oncocytomas (**Figure 7D**) are isoechoic or hypoechoic to the renal parenchyma. It is difficult for the naked eye to identify oncocytomas. In this study, some cases of AMLs and oncocytomas were incorrectly described by sonographers as malignant masses. Thus, the overall diagnostic accuracy was lower in the sonographers than in the radiomics model. However, the sensitivity of the radiomics model was lower than that of the senior and junior physicians. In the validation set, 10 renal malignancies were mistaken as benign masses by the radiomics model. Six of the 10 cases were chromophobe RCC. Both chromophobe RCC and renal oncocytoma originate from intercalated cells of the collecting duct system and shared some overlapping morphologic, histochemical, immunohistochemical, and ultrastructural features (36, 37). This may be the reason for the incorrect diagnosis.

Unexpectedly, in this study, the radiomics nomogram model in combination with the clinical factor did not show better performance compared with the radiomics model alone as shown in the study of Nie et al. (24). Their nomogram incorporating the CT-based radiomics and clinical factors outperformed the radiomics signature alone for differentiating AML from clear cell RCC. We propose that the discrepancies may be due to the sample size and pathological subtypes. In this study, a large cohort of 600 patients was analyzed and more pathological subtypes were included, except for AML and clear cell RCC. Sex was the only clinical signature in the nomogram of this study, suggesting that clinical factors in renal masses may contribute minimally to the differentiation of benign from malignant renal masses as reported in other cancers including ovarian, breast, and lung cancers (38–40). This observation merits further investigation.

It is noteworthy to point out the limitations of this study. First, its retrospective nature poses a potential selection bias. The US images used in this study were derived from two different US machines. This may lead to heterogeneity of the US images and further impact the performance of the radiomics score. Second, the samples were derived from a single institute, and the training and validation cohorts were split using a random split method. External validation using a prospectively recruited patient cohort and multicenter studies with a larger sample size to prove the robustness of our results are required. Third, manual region of interest (ROI) segmentation is time-consuming and complicated, especially for tumors without a well-defined boundary. Future studies should focus on the development of an automatic segmentation method for renal tumors with favorable reliability and reproducibility.

## Conclusion

In conclusion, this study developed an ultrasonic–radiomic nomogram model that showed favorable predictive efficacy in preoperatively distinguishing benign renal masses from malignant tumors. As a non-invasive and quantitative method, the radiomics nomogram model may serve as an effective tool to supplement conventional imaging modalities for the clinical decision-making process.

## Data Availability Statement

The original contributions presented in the study are included in the article/**Supplementary Material**. Further inquiries can be directed to the corresponding authors.

## Ethics Statement

This study was reviewed and approved by the Ethics Committee of Tianjin Medical University Cancer Institute and Hospital (Approval No. bc2021124). The Ethics Committee waived the requirement of written informed consent for participation.

## Author Contributions

WM and CL designed the study. CL and GQ wrote the original draft. JL, LQ, and XQW performed the analyses. TZ, XL, and SD collected the data. WM and XW contributed to the discussion and manuscript revision. All authors contributed to the article and approved the submitted version.

## Funding

This study was supported by the scientific research program of Tianjin Education Commission (2020KJ131).

## Conflict of Interest

The authors declare that the research was conducted in the absence of any commercial or financial relationships that could be construed as a potential conflict of interest.

## Publisher’s Note

All claims expressed in this article are solely those of the authors and do not necessarily represent those of their affiliated organizations, or those of the publisher, the editors and the reviewers. Any product that may be evaluated in this article, or claim that may be made by its manufacturer, is not guaranteed or endorsed by the publisher.
