# Corrigendum: An ultrasonic-based radiomics nomogram for distinguishing between benign and malignant solid renal masses

**DOI:** 10.3389/fonc.2023.1232714

**Published:** 2023-06-14

**Authors:** Chunxiang Li, Ge Qiao, Jinghan Li, Lisha Qi, Xueqing Wei, Tan Zhang, Xing Li, Shu Deng, Xi Wei, Wenjuan Ma

**Affiliations:** ^1^ Department of Diagnostic and Therapeutic Ultrasonography, Tianjin Medical University Cancer Institute and Hospital, Tianjin, China; ^2^ National Clinical Research Center for Cancer, Tianjin, China; ^3^ Key Laboratory of Cancer Prevention and Therapy, Tianjin, China; ^4^ Tianjin’s Clinical Research Center for Cancer, Tianjin, China; ^5^ Department of Pathology, Tianjin Medical University Cancer Institute and Hospital, Tianjin, China; ^6^ Department of Diagnostic and Therapeutic Ultrasonography, Tianjin Ninghe Hospital, Tianjin, China; ^7^ Second Hospital of Tianjin Medical University, Tianjin, China; ^8^ Department of Breast Imaging, Tianjin Medical University Cancer Institute and Hospital, Tianjin, China

**Keywords:** renal mass, angiomyolipoma, oncocytoma, ultrasound, radiomics

In the published article, there was an error regarding the affiliations for Ge Qiao. As well as having affiliations 2,3,4,5, they should also have affiliation 1 (Department of Diagnostic and Therapeutic Ultrasonography, Tianjin Medical University Cancer Institute and Hospital, Tianjin, China).

The authors apologize for this error and state that this does not change the scientific conclusions of the article in any way. The original article has been updated.

