# Supplementary material for: An Ultrasonic-Based Radiomics Nomogram for Distinguishing Between Benign and Malignant Solid Renal Masses
Source: Front Oncol. 2022 Mar 4;12:847805. doi: 10.3389/fonc.2022.847805 (PMC8931199; doi:10.3389/fonc.2022.847805)
Supplement: Supplementary file 1 [file Table_1.docx]

Supplementary Material

# Study Cohort and Imaging Dataset

**Table S1 Summary of patient characteristics**

| characteristics | Malignant lesions (n=396) | Benign lesions (n=204) |
| --- | --- | --- |
| Mean age(y) | 55.2±10.8 | 49.7±12.0 |
| Gender (male/female) | 265/131 | 41/163 |
| Symptoms(yes/no) | 71/325 | 32/172 |
| Location(left/right) | 191/205 | 108/96 |
| Mean diameter_max_ | 4.89±2.34 | 5.02±3.16 |
| Operation(Radical/Partial nephrectomy ) | 229/167 | 55/149 |
| Pathological type | Clear cell RCC 301(76.0%) | AMLs 170(83.3%)  Oncocytomas 29(14.2%) |
|  | Chromophobe RCC 44(11.1%) |  |
|  | Papillary RCC 41(10.4%) | Other types 5(2.4%) |
|  | Other types 10(2.5%) |  |

Other type RCCs: three clear cell papillary RCC, two Multilocular cystic RCC, one Pigmented Xp11 translocation RCC, one Unclassified RCC, one Myxoid tubular carcinoma, one Translocation of RCC in accordance with Mit(6;11) and one Collecting duct carcinoma. Other type benign lesions: three metanephric adenomas, one renal leiomyoma and one renal fibroma

1. **US image feature extraction**

To normalize the different image specifications from various US scanners, image resampling and gray-level normalization were performed before quantitative feature extraction. All image data were resampled at a 1×1-mm voxel space size. The quantitative features were extracted from ROIs using an in-house software developed with MATLAB 2018B (The MathWorks Inc, Natick, MA, USA)

A total of 855 radiomic features were drawn from each segmented lesion and can grouped as follows: (1) Morphologic features: four metrics, including area, largest diameter, length to width ratio and roundness, were calculated for the morphological description of the images. Area is the number of voxels in the tumor region extracted from US images multiplied by the dimension of voxels. Largest diameter is the voxels number of the long axis. Length to width ratio is the ratio of length to width. Roundness is defined as the ratio of the circumcircle radius to the inscribed circle radius of the lesion ROI. (2) Gray-scale histograms features: three features were computed for each lesion according to the definitions of the gray-scale histogram: variance, skewness and kurtosis. Their definition can be found in literatures Rodenacker K (2003). (3) Texture features: in total, 30 texture features were extracted from the tumor regions of US images after wavelet transform. Detailed description and methodology employed to extract the texture features is available in M Vallières (2015). (4) Wavelet features: wavelet transform effectively decouples textural information by decomposing the original image. In this study a discrete, one-level and undecimated two dimensional wavelet transform was applied to each US image, which decomposes the original image into 4 decompositions (LL, HL, LH and HH). For each decomposition we computed gray-scale histograms and the textural features as described in Table S2.

**Table S2. Texture features used in this study.**

| **Texture type** | **Reference(s)** | **Texture name** |
| --- | --- | --- |
| **GLCM**^a^ | (Haralick et al 1973) | Energy |
|  |  | Contrast |
|  |  | Correlation |
|  |  | Homogeneity |
|  |  | Variance |
|  |  | Sum Average |
|  |  | Entropy |
|  |  | Dissimilarity |
|  |  | Auto Correlation |
| **GLRLM**^b^ | (Galloway 1975) | Short Run Emphasis (SRE) |
|  |  | Long Run Emphasis (LRE) |
|  |  | Gray-Level Non-uniformity (GLN) |
|  |  | Run-Length Non-uniformity (RLN) |
|  |  | Run Percentage (RP) |
|  | (Chu et al 1990) | Low Gray-Level Run Emphasis (LGRE) |
|  |  | High Gray-Level Run Emphasis (HGRE) |
|  | (Dasarathy and Holder 1991) | Short Run Low Gray-Level Emphasis (SRLGE) |
|  |  | Short Run High Gray-Level Emphasis (SRHGE) |
|  |  | Long Run Low Gray-Level Emphasis (LRLGE) |
|  |  | Long Run High Gray-Level Emphasis (LRHGE) |
|  | (Thibault et al 2009) | Gray-Level Variance (GLV) |
|  |  | Run-Length Variance (RLV) |
| **GLSZM**^c^ | (Galloway 1975, Thibault et al 2009) | Small Zone Emphasis (SZE) |
|  |  | Large Zone Emphasis (LZE) |
|  |  | Gray-Level Non-uniformity (GLN) |
|  |  | Zone-Size Non-uniformity (ZSN) |
|  |  | Zone Percentage (ZP) |
|  | (Chu et al 1990, Thibault et al 2009) | Low Gray-Level Zone Emphasis (LGZE) |
|  |  | High Gray-Level Zone Emphasis (HGZE) |
|  | (Dasarathy and Holder 1991, | Small Zone Low Gray-Level Emphasis (SZLGE) |
|  | Thibault et al 2009) | Small Zone High Gray-Level Emphasis (SZHGE) |
|  |  | Large Zone Low Gray-Level Emphasis (LZLGE) |
|  |  | Large Zone High Gray-Level Emphasis (LZHGE) |
|  | (Thibault et al 2009) | Gray-Level Variance (GLV) |
|  |  | Zone-Size Variance (ZSV) |
| **NGTDM**^d^ | (Amadasun and King 1989) | Coarseness |
|  |  | Contrast |
|  |  | Busyness |
|  |  | Complexity |
|  |  | Strength |

^a^ GLCM: Gray-level co-occurrence matrix.

^b^ GLRLM: Gray-level run-length matrix.

^c^ GLSZM: Gray-level size zone matrix.

^d^ NGTDM: Neighborhood gray-tone difference matrix.

1. **Radiomics score**

RadScore was calculated by summing the selected features weighted by their coefficients. The final formula of rad-score is:

*Radscore=1.373119859-0.495535038*text_Ng_8_vox_1_glszm_ZSN-0.116202160*text_Ng_8_vox_1_glszm_GLV-0.181637246*text_Ng_16_vox_1_glszm_SZLGE*

*+0.194220148*text_Ng_32_vox_1_glszm_ZSV+0.030519617*text_Ng_64_vox_1_ngtdm_Complexity-0.057947353*wave_glszm_8_LL_ZSN-0.266823009*wave_glszm_8_LL_SZHGE+0.024134030*wave_glszm_8_HL_LZLGE+0.199924975*wave_glrlm_8_LH_RLV-0.479845518*wave_ngtdm_8_LH_Coarseness-0.133563329*wave_ngtdm_8_HH_Strength-0.233596752*wave_glszm_16_LL_SZE-1.556806924*wave_glcm_16_HL_Correlation-0.149557240*wave_glrlm_16_HL_RLV-0.020992385*wave_glszm_16_HL_ZSV+0.076258787*wave_glrlm_16_LH_RLV+0.189142597*wave_ngtdm_16_LH_Complexity-0.060455660*wave_glrlm_16_HH_LRHGE+0.217729414*wave_glrlm_16_HH_GLV-0.003893129*wave_glszm_16_HH_SZE+0.079545181*wave_glrlm_32_LH_GLN+0.118917950*wave_glrlm_32_LH_RLV-0.213789098*wave_glcm_32_HH_AutoCorrelation-0.756742442*wave_glszm_64_LL_LZHGE-0.296399406*wave_glszm_64_HL_LZHGE+0.048347709*wave_glszm_64_HL_ZSV+0.104968327*wave_glo_64_LH_Kurtosis-0.315588955*wave_glcm_64_HH_Correlation+0.054442734*wave_glrlm_64_HH_RLV*
